# Serum Uric Acid Is Positively Associated with Muscle Mass and Strength, but Not with Functional Capacity, in Kidney Transplant Patients

**DOI:** 10.3390/nu12082390

**Published:** 2020-08-10

**Authors:** João Pedro Floriano, Paula C. Nahas, Flávia M. S. de Branco, Aline S. dos Reis, Luana T. Rossato, Heitor O. Santos, Larissa S. Limirio, Sebastião R. Ferreira-Filho, Erick P. de Oliveira

**Affiliations:** 1 Laboratory of Nutrition, Exercise and Health (LaNES), School of Medicine, Federal University of Uberlandia (UFU), Uberlandia, Minas Gerais 38400-902, Brazil; floriano.unesp@gmail.com (J.P.F.); nahaspaula6@gmail.com (P.C.N.); fla-msb@hotmail.com (F.M.S.d.B.); alinereis14@hotmail.com (A.S.d.R.); luanathrossato@hotmail.com (L.T.R.); heitor13cam@hotmail.com (H.O.S.); larissa-limirio@hotmail.com (L.S.L.); 2 School of Medicine, Federal University of Uberlandia (UFU), Uberlandia, Minas Gerais 38400-902, Brazil; sebahferreira@gmail.com

**Keywords:** uric acid, sarcopenia, muscle function, renal patients

## Abstract

Background: Our aim was to associate serum uric acid (UA) with muscle mass, strength and functional capacity in kidney transplant patients (KTPs). Methods: A cross-sectional study was performed on 113 KTPs. The fat-free mass and total and appendicular muscle mass were estimated by performing a bioelectrical impedance analysis. The strength was evaluated using the handgrip strength test (HGS) and the five times sit to stand test (5STS). The functional capacity was evaluated using the 4 m walk test and the short physical performance battery (SPPB). Results: Linear regression showed that the UA levels were positively associated with the muscle mass, fat-free mass, appendicular muscle mass, muscle mass index and appendicular muscle mass index. The 5STS results (seconds) were inversely associated with the UA levels, showing that individuals with higher UA were more likely to have more strength. However, UA was not associated with the HGS, 4 m walk test and SPPB results. Conclusion: UA levels were positively associated with muscle mass and strength, but not with functional capacity, in KTPs.

## 1. Introduction

Chronic kidney patients may present decreased muscle mass, strength and functional capacity [1], which impacts their general health [2] and increases the risk of mortality [3]. Recently, our research group showed that ~19% of kidney transplant patients (KTPs) attended an outpatient clinic presenting with sarcopenia [4], which is defined as a loss of strength, decreased muscle mass, and low functional capacity [5]. For individuals without kidney disease, the main causes of muscle mass and strength losses are aging, hormonal changes, increased inflammation, lack of physical activity and low protein intake [6]. However, individuals with chronic kidney disease present additional causes for muscle depletion, such as uremia, medicine use (with proteolytic action [7]), metabolic acidosis, a loss of nutrients in dialysis, and increased oxidative stress [8].

Indeed, KTPs may have an impaired glomerular filtration rate, which decreases uric acid (UA) excretion, increasing the serum UA levels [9]. Additionally, UA is the end product of purine metabolism in humans and is a powerful antioxidant―responsible for more than half of the total plasma antioxidant capacity [10]. In this regard, the increased UA levels that are commonly observed in KTPs would have an effect on muscle mass because oxidative stress seems to be one of the causes of muscle mass and strength loss [11,12]. Nevertheless, to date, the associations that link serum UA levels to muscle mass and strength loss have not been evaluated in KTPs.

Several studies have associated UA with muscle mass, strength or functional capacity in non-KTPs [13,14,15,16,17,18,19,20,21,22]. The relationship between UA and muscle mass is unclear because positive associations were observed in healthy Asian individuals [16] and in hemodialysis Israeli patients [14], whereas negative associations were noted in the American population [13] and in Brazilian individuals with a moderate prevalence of Metabolic Syndrome [15]. Regarding the associations between UA and muscle strength/function, the conclusions are also unclear. Huang et al. [20] showed that muscle strength was lower in middle-aged men with high plasma UA levels. Controversially, other studies showed a positive association between muscle strength and hyperuricemia in older adults [17,18,19,21,22]. Collectively, these results show that it is still unclear whether UA is positively associated with muscle mass, strength and muscle function in non-KTPs. Considering that KTPs may present increased UA levels due to decreased glomerular filtration and medicine use [23,24] (and can have different causes for the loss of muscle mass and strength, compared with the non-renal disease population), the associations of UA with muscle mass, strength and muscle function are unknown in this population. Therefore, the aim of this study was to associate serum UA levels with the muscle mass, strength and functional capacity in KTPs.

## 2. Methods

### 2.1. Participants

A cross-sectional study was conducted at the Hospital of the Federal University of Uberlandia (Minas Gerais, Brazil), which has a kidney disease ambulatory clinic. The inclusion criteria were subjects older than 18 years with at least 3 months of kidney transplantation, who were able to answer the questions and perform the physical tests. The exclusion criteria were kidney graft rejection and/or failure and KTPs on dialysis. In total, 360 subjects attended the Kidney Disease Ambulatory; 113 volunteers were included in the study (Figure S1). This research was approved by the Federal University of Uberlandia Research Ethics Committees (protocol number: 1688246), and all participants signed a consent form.

### 2.2. Anthropometric Assessment

The body weight was measured with a portable digital scale (Líder^®^), and the height, by a vertical mobile stadiometer (Welmy^®^). After obtaining body weight and height measurements, the body mass index (BMI) was calculated. The waist circumference was measured with a non-elastic tape (Cescorf^®^), which was positioned at the midpoint between the last rib and the iliac crest [25]. The mean from the three measurements was used.

### 2.3. Dietary Assessment

The dietary assessment was performed by 24 h dietary recalls on two different occasions―one, a face-to-face interview and the other, a phone call (4 to 10 days after the first dietary recall) [26]. A trained nutritionist interviewed each volunteer following the 5-step multiple pass method [27]. The data collected were analyzed and calculated by the Nutrition Data System for Research (NDS-R^©^), software version 2014.

### 2.4. Physical Activity Level

The short version of International Physical Activity Questionnaires (IPAQ) was applied to determine the physical activity level [28]. This questionnaire was validated for the Brazilian population and provided knowledge about the duration, frequency and intensity of physical activities performed in the last week.

### 2.5. Body Composition

Body composition was evaluated using bioimpedance (Biodynamics^®^ 450, Seattle, WA, USA) with a frequency of 50 kHz. To ensure their adequate hydration, the participants were asked to avoid consuming caffeine and alcoholic beverages and not to perform intense physical activity one day before the test. The participants were evaluated after a 12 h overnight fast. The participants were advised to empty their urinary bladders 30 min prior to the test and were instructed to remain in the supine position for five minutes to ensure their body fluids balanced. The HeartBeat (HeartBeat, Biotronik Comercial Médica Ltd., São Paulo, Brazil) electrodes were positioned on the right side of the body at the wrist, hand, ankle and foot, after each site was cleaned with alcohol. Values between 69 and 75% of the total body water per lean mass were considered acceptable for a reliable bioimpedance test [29]. For the women of childbearing age, the evaluation was conducted outside of their menstrual periods.

We used the raw bioimpedance data to estimate the body fat, fat-free mass and total body water. Janssen et al.’s equation [30] was used to estimate the total muscle mass in kilograms. The muscle mass index was calculated, which consisted of the muscle mass (in kilograms) divided by the square of the height (in meters). The appendicular skeletal muscle mass was calculated following the new sarcopenia consensus recommendations [5], using Sergi et al.’s equation [31]. The appendicular skeletal muscle mass index equation was adopted (Appendicular skeletal muscle mass (kg)/Height (m^2^)) [5].

### 2.6. Strength and Functional Capacity

The handgrip strength (HGS) test and the five times sit to stand test (5STS) were performed to assess the muscle strength [5]. The HGS was measured three times by the use of the dominant hand in a hydraulic dynamometer Jamar^®^. Each subject was seated with their arm in a neutral rotation and a flexed elbow at 90°, in order that the dynamometer could be squeezed with maximum power, and the highest value was considered. Regarding 5STS, the participant was instructed to sit and get up from the chair five times, as fast as possible―with this test time being recorded [1,5].

The functional capacity was evaluated using the short physical performance battery (SPPB) and the 4 m walk speed test. The SPPB included balance tests, walk speed and the 5STS, assessed together. Each test had 4 points maximum, which were totalized as 12 points by the end of the test [32]. The balance test has the purpose of evaluating if the participants can stay in three positions for ten seconds each: the feet together, the semi-tandem position and the tandem. The 4 m walk test consists of: 1 m to the acceleration zone, 4 m in which the subject should walk at the usual gait speed they achieve during their daily activities, and 1 m to the deceleration zone. The walk test was repeated without rest, and the attempt performed in a shorter time was used to calculate the speed in meters per second [33]. All subjects received a voice command from the evaluator to start the test.

### 2.7. Blood Sample Analysis

The blood samples were collected after a 12 h overnight fast, on the same day that the bioimpedance was performed. The electrochemiluminescence method was employed to analyze the plasma levels of creatinine, urea, C-Reactive Protein, glucose, triglycerides, and total and fraction cholesterol levels. Low density lipoprotein (LDL-c) was calculated using the Friedewald equation. The glomerular filtration rate was estimated by the chronic kidney disease epidemiology collaboration (CKD-EPI) equation [34]. The tacrolimus and cyclosporine levels were measured using micro-particle-based immune aggregation. The enzymatic colorimetric method was used to assess the UA serum levels. The cutoff point to classify elevated UA was > 7.0 mg/dL for men and UA > 6.0 mg/dL for women [10].

### 2.8. Statistical Analysis

The participants were characterized according to the UA classification (elevated vs. normal). For the continuous variables, the t-Student test or Mann–Whitney test was performed, and the data were described as mean and standard deviation, or median, minimum and maximum. The chi-squared test was used to compare the data in percentages (the categorical variables). A multiple linear regression model was performed to associate the UA levels with the muscle mass, strength, and functional capacity. Uric acid (the independent variable) was inserted in the model, with the confounder’s variables, to evaluate the prediction of the variances of the muscle mass, strength or functional capacity (the dependent variables). The R^2^ value of each statistical model was generated, and then, a second analysis was performed, removing the UA from the model. The difference between the R^2^ values of the two models was used to estimate the prediction of muscle mass, strength or functional capacity values by the UA levels in an isolated form. The confounders added in the statistical model were sex, age, physical activity level, protein intake (g/kg), glomerular filtration rate, allopurinol use and waist circumference. These analyses were also performed by evaluating men and women separately. For the total sample, we performed an additional linear regression analysis adjusting for the following: sex, age, physical activity, protein intake, glomerular filtration rate, allopurinol use, waist circumference, triglyceride levels, diabetes, hypertension, tacrolimus and cyclosporine blood levels, smoking status and loop diuretic use (Table S1). Stata 14 (StataCorp, College Station, TX, USA) was used to perform the analysis. A *p* value ≤ 0.05 was considered as significant.

## 3. Results

The participants with the elevated UA levels presented higher values of triglycerides, urea and creatinine levels. They used higher doses of loop diuretics (18.7 ± 24.5 vs. 8.6 ± 20.1 mg/day) and had a lower glomerular filtration rate than those individuals with normal UA. No difference was observed between the groups in terms of demographics, physical activity, anthropometric measurements, body composition, physical performance, strength, medicine use, tacrolimus and cyclosporine blood levels, kidney transplantation and biochemical parameters, and dietary intake (Table 1). The use of drugs is also described as mean values in Table S3.

The multiple linear regression showed that the UA levels were positively associated with the muscle mass, fat-free mass, appendicular muscle mass, muscle mass index and appendicular muscle mass index. The UA predicted the variances of these muscle parameters by 1.58–3.61%. The 5STS (s) was inversely associated with the UA levels, showing that individuals with higher UA were more likely to have more strength. However, the UA was not associated with the HGS, 4 m walk test and SPPB scores (Table 2). All these associations remained the same after additional adjustments were made for diabetes, triglyceride levels, hypertension, tacrolimus and cyclosporine blood levels, smoking status and loop diuretic use (Table S1).

In a sub-analysis, which evaluated men and women separately, the UA was associated with the muscle mass and 5STS results (s) in men (as was observed for the total sample); whereas no significant associations were observed for women (Table S2).

## 4. Discussion

We found that the serum UA levels were positively associated with the muscle mass in the KTPs. The linear regression analyses showed that UA levels explained the variances of fat-free/muscle mass by ~1.5–3.6%. In addition, the UA was positively associated with strength when it was evaluated by 5STS, but no association was observed when it was assessed by the HGS. Collectively, these results suggest that UA seems to be a factor in protecting the muscle mass and the strength of the lower limbs in KTPs.

In a sub-analysis, which was separated by sex, the UA was associated with the muscle mass and strength in men, but not in women, which suggests that these associations can be sex-specific. However, we cannot ignore the fact that the limited number of women who were evaluated in the present study could also explain the absence of this association. Future studies should be performed to evaluate a higher number of women, in order to confirm whether the association between UA and muscle mass/strength in KTPs is sex-dependent.

The exact mechanism that explains the positive associations of UA with muscle mass and strength in KTPs remains unclear, but it is possible to suggest that it could be related to the antioxidant properties of UA [10]. Particularly in KTPs, oxidative stress is higher than in other populations (for example, healthy individuals) [8], and the excess of reactive oxygen and nitrogen species seems to affect muscle size, fiber activation and excitation–contraction [11]. In this way, the KTPs with higher UA levels could have greater protection against the excesses of reactive oxygen species, preserving their higher muscle mass and strength.

We did not observe an association between UA and functional capacity. Although the exact mechanism is unknown, it can be possibly explained due to the age range of the KTPs evaluated in the present study. Most of the KTPs were middle-aged individuals (mean age value ~47 y) who had a normal functional capacity. Although the decreases in functional capacity begin at approximately the age of 40 y, important decreases in functional capacity occur mainly in individuals aged 70 y or more [35]. Elevated oxidative stress is associated with a low gait speed, but this association is observed mainly in older adults [36]. In middle-aged individuals, such as most of the participants in the present study, the effects of increased oxidative stress on the decreases in gait speed are in their infancy. Therefore, these individuals probably have sufficient reserve capacity for short-term walking, despite the excessive oxidative stress induced by renal disease. Thus, future studies that associate UA and functional capacity exclusively in older adults with renal disease should be performed.

The present study has limitations. We evaluated body composition using bioimpedance; the muscle mass was estimated using equations that were not validated for KTPs. However, to minimize this limitation, we included several forms (and equations) to estimate muscle/fat-free mass; the linear associations were statistically significant for all the muscle variables. It shows that although there is not a valid equation that can estimate the muscle mass of KTPs, our results are trustworthy. In addition, the cross-sectional design does not allow us to determine the cause–effect relationship. As for strength, this research is the first to report a positive association between the UA levels and the muscle mass/strength in KTPs. Elevated UA is usually considered a risk factor for the progression of reduced kidney function and the loss of transplantation [37], and, therefore, drug interventions are often performed to reduce the UA levels; however, these deleterious effects are not a consensus [38,39]. Thus, the results of the present study may have important clinical implications because we showed that UA can be a potential protective factor for muscle mass and strength in KTPs. Future randomized clinical trials should be performed, in order to investigate the possible causal effect of UA on the muscle mass and strength in this population. This may show that it would not be beneficial to decrease the UA levels in KTPs, at least where muscle mass is concerned.

In conclusion, UA levels were positively associated with muscle mass and strength, but not with functional capacity, in KTPs.

## Figures and Tables

**Table 1:** Characteristics of the participants according to uric acid levels.

	Normal Uric Acid (*n* = 66)	Elevated Uric Acid (*n* = 47)	*p*-Value
Uric Acid (mg/dL) *	5.4 (3.1–7.0)	7.9 (6.1–13.4)	<0.001
**Demographic Parameters and Physical Activity**			
Age (y)	47.9 ± 12.4	47.4 ± 12.6	0.841
Sex (men/women) (n)	45/21	30/17	0.629
Physical activity (min/week) *	145 (0.0–780)	80 (0.0–840)	0.446
**Anthropometric Parameters**			
Height (m)	1.63 ± 0.08	1.64 ± 0.09	0.684
Weight (kg)	68.3 ± 15.2	73.9 ± 14.2	0.052
Body mass index (kg/m^2^)	25.6 ± 5.6	27.6 ± 5.3	0.060
Waist circumference (cm) *	90.1 (63.0–179)	93.1 (73.5–135)	0.117
**Body Composition**			
Total body water (liter)	35.8 ± 6.5	38.0 ± 6.5	0.073
Total body water/lean mass (liter/kg) *	72.1 (69.7–75.3)	72.0 (69.4–75.8)	0.224
Muscle mass (kg) *	25.3 (15.3–34.2)	27.0 (12.9–36.0)	0.322
Fat-free mass (kg)	49.3 ± 9.3	52.6 ± 9.1	0.058
Appendicular skeletal muscle mass (kg)	18.8 ± 3.4	19.8 ± 3.6	0.115
Fat mass (kg) *	17.2 (5.2–47.5)	19.2 (9.4–50.1)	0.210
Fat mass (%)	27.0 ± 8.6	28.2 ± 7.8	0.443
Muscle mass index (kg/m^2^)	9.12 ± 1.43	9.35 ± 1.67	0.442
Appendicular skeletal muscle mass index (kg/m^2^)	7.00 ± 0.94	7.30 ± 1.01	0.120
**Physical Performance and Strength**			
Short physical performance battery * (score)	11.0 (7.0–12.0)	11.0 (2.0–12.0)	0.960
4 m walking test (m/s)	1.1 ± 0.2	1.1 ± 0.3	0.214
Five times sit to stand test (s) *	11.1 (7.5–31.6)	10.7 (7.4–16.9)	0.222
Handgrip strength (kg) *	14.5 (4.0–40.0)	16.0 (4.0–62.0)	0.436
**Drugs**			
Allopurinol (mg/day) *	0.0 (0.0–200)	0.0 (0.0–200)	0.260
Prednisone (mg/day) *	5.0 (0.0–10.0)	5.0 (0.0–50.0)	0.839
Tacrolimus (mg/day) *	2.0 (0.0–16.0)	2.0 (0.0–10.0)	0.447
Tacrolimus blood levels (ng/mL) *	3.5 (0.0–19.8)	4.0 (0.0–20.9)	0.835
Cyclosporine (mg/day) *	0.0 (0.0–150)	0.0 (0.0–200)	0.701
Cyclosporine blood levels (ng/mL) *	0.0 (0.0–411)	0.0 (0.0–921)	0.980
Everolimus (mg/day) *	0.0 (0.0–2.0)	0.0 (0.0–1.5)	0.745
Sirolimus (mg/day) *	0.0 (0.0–2.0)	0.0 (0.0–0.0)	0.140
Azathioprine (mg/day) *	0.0 (0.0–100)	0.0 (0.0–100)	0.202
Mycophenolate sodium (mg/day) *	0.0 (0.0–1440)	0.0 (0.0–1440)	0.546
Mycophenolate mofetil (mg/day) *	0.0 (0.0–2000)	0.0 (0.0–2000)	0.829
Loop diuretics (mg/day) *	0.0 (0.0–80.0)	0.0 (0.0–80.0)	0.008
Thiazide diuretics (mg/day) *	0.0 (0.0–50.0)	0.0 (0.0–50.0)	0.399
Corticoids drugs, n (%)	61 (92.4)	42 (89.4)	0.572
Calcineurin inhibitor use, ** n (%)	44 (66.7)	31 (66.0)	0.937
Cell proliferation inhibitor use, † n (%)	57 (86.4)	39 (83.0)	0.850
mTOR inhibitor use, ‡ n (%)	13 (19.7)	8 (17.0)	0.719
Loop diuretic use, § n (%)	13 (19.7)	20 (42.5)	0.008
Thiazide diuretic use, ¶ n (%)	2 (3.0)	3 (6.4)	0.393
**Kidney Transplantation Data**			
Urea (mg/dL) *	39.6 (15.0–141.2)	50.2 (24.9–156.5)	<0.001
Creatinine (mg/dL) *	1.3 (0.7–6.1)	1.6 (0.8–8.7)	<0.001
Glomerular filtration rate CKD-EPI (ml/min/1.73) m^2^)	61.8 ± 20.6	45.8 ± 19.6	<0.001
C-reactive protein (mg/dL) *	0.3 (0.03–22.4)	0.3 (0.03–11.7)	0.546
Time since transplantation (months) *	66.0 (3.0–336)	74.0 (8.0–444)	0.893
Pre-transplant body mass index (kg/m^2^) *	21.7 (15.6–33.2)	23.0 (17.7–34.6)	0.290
Dialysis time before transplantation (months) *	36.0 (4.0–195)	36.0 (5.0–192)	0.741
*Type of dialysis before transplant n (%)*			
Peritoneal dialysis	4 (6.1%)	3 (6.4%)	0.349
Hemodialysis	54 (81.8%)	42 (89.4%)	
Peritoneal dialysis and hemodialysis	8 (12.1%)	2 (4.3%)	
*Type of Donor n (%)*			
Living	23 (34.8%)	19 (40.4%)	0.545
Deceased	43 (65.2%)	28 (59.6%)	
1st Transplant/2nd Transplant (n)	61/5	42/5	0.572
**Biochemical Parameters**			
Glucose (mg/dL) *	92.0 (70.0–407)	94.0 (63.0–193)	0.894
Triglycerides (mg/dL) *	148.1 (43.0–618)	165.5 (93.5–715)	0.040
Total cholesterol (mg/dL)	185.1 ± 45.9	189.7 ± 37.6	0.571
HDL cholesterol (mg/dL) *	48.0 (24.0–87.4)	44.0 (24.0–99.0)	0.244
LDL cholesterol (mg/dL) *	101 (26.0–239)	102 (39.0–210)	0.917
**Health Conditions and Habits**			
Hypertension n (%)	52 (78.8)	39 (83.0)	0.579
Diabetes n (%)	16 (24.2)	10 (21.3)	0.712
Smoking/non-smoking (n)	4/62	2/45	0.673
**Dietary Assessment**			
Energy (kcal) *	1600 (592–2862)	1575 (924–3021)	0.904
Carbohydrates (g) *	215 (77.2–426)	210 (49.6–353)	0.459
Protein (g) *	78.1 (30.6–196)	72.1 (22.8–174)	0.843
Protein (g/kg) *	1.14 (0.4–3.4)	1.10 (0.3–4.8)	0.875
Fat (g) *	60.2 (18.0–124)	48.3 (23.4–132)	0.193

Notes: CKD-EPI, chronic kidney disease epidemiology collaboration equation; HDL, high density lipoprotein; LDL, low density lipoprotein; * Non-parametric data. Data described as the median and minimum and maximum; ** Calcineurin inhibitor: tacrolimus or cyclosporine; † Cell proliferation inhibitors: azathioprine, mycophenolate sodium and mycophenolate mofetil; ‡ mTOR inhibitors: everolimus and sirolimus; § Loop diuretics: furosemide; ¶ Thiazide and thiazide-like diuretics: hydrochlorothiazide and chlorthalidone.

**Table 2:** Linear regression analysis of uric acid with muscle mass, strength and functional capacity.

	β (Uric Acid Value)	R^2^ %	* R^2^ %	*p*-Value
Fat-free mass (kg)	0.219	70.72	3.43	<0.001
Muscle mass (kg)	0.160	76.88	1.84	0.005
Appendicular skeletal muscle mass (kg)	0.224	73.99	3.61	<0.001
Muscle mass index (kg/m^2^)	0.149	67.34	1.58	0.027
Appendicular skeletal muscle mass index (kg/m^2^)	0.220	69.33	3.49	<0.001
Short physical performance battery (score)	0.108	29.09	0.84	0.270
4 m walk test (m/s)	−0.129	20.11	1.19	0.216
Handgrip strength (kg)	0.147	42.64	1.55	0.097
Five times sit to stand test (s)	−0.245	15.79	4.31	0.023

Notes: Adjusted for sex, age, physical activity, protein (g/kg), glomerular filtration rate, allopurinol and waist circumference; * R^2^% = R^2^ value of the uric acid plus adjustments minus the R^2^ value of the statistical model with only the adjustments variables.

## Supplementary Materials

The following are available online at https://www.mdpi.com/2072-6643/12/8/2390/s1. Figure S1: Flow-chart of the participants, Table S1: Linear regression analysis of uric acid with muscle mass, strength, and functional capacity, Table S2: Linear regression analysis of uric acid with muscle mass, strength, and functional capacity according to sex, Table S3: Characteristics of the participants according to uric acid levels.
