# Supplementary material for: Serum Uric Acid Is Positively Associated with Muscle Mass and Strength, but Not with Functional Capacity, in Kidney Transplant Patients
Source: Nutrients. 2020 Aug 10;12(8):2390. doi: 10.3390/nu12082390 (PMC7469022; doi:10.3390/nu12082390)
Supplement: Supplementary file 1 [file nutrients-12-02390-s001.pdf]

Supplemental table 1. Linear regression analysis of uric acid with muscle mass, strength, and functional capacity.

	**β (uric acid value)**	***p-value***
Fat-Free Mass (kg)	1.081	**0.001**
Muscle Mass (kg)	0.462	**0.006**
Appendicular Skeletal Muscle Mass (kg)	0.445	**< 0.001**
Muscle Mass Index (kg/m²)	0.110	**0.050**
Appendicular Skeletal Muscle Index (kg/m²)	0.118	**0.001**
Short Physical Performance Battery (score)	0.089	0.290
4-meter walk test (m/s)	-0.012	0.348
Handgrip Strength (kg)	0.826	0.062
Five times sit to stand test (s)	-0.464	**0.035**

Notes: Adjusted for sex, age, physical activity, protein intake (g/kg), glomerular filtration rate, allopurinol use, waist circumference, triglyceride levels, diabetes, hypertension, tacrolimus and cyclosporine blood levels, smoking status and loop diuretics use.

Supplemental Table 2. Linear regression analysis of uric acid with muscle mass, strength, and functional capacity according to sex.

	Simple linear regression		Multiple linear regression			
	β (uric acid)	*p-value*	β (uric acid)	R^2 %	*R^2 %	*p-value*
Men (n=75)						
Fat-Free Mass (kg)	1.565	**0.001**	0.934	63.47	3.77	**0.011**
Muscle Mass (kg)	0.627	**0.001**	0.516	33.15	6.92	**0.010**
Appendicular Skeletal Muscle Mass (kg)	0.588	**<0.001**	0.387	56.77	5.44	**0.005**
Muscle Mass Index (kg/m²)	0.175	**0.005**	0.141	31.01	5.08	**0.030**
Appendicular Skeletal Muscle Index (kg/m²)	0.588	**<0.001**	0.105	63.52	3.99	**0.009**
Short Physical Performance Battery (score)	-0.017	0.828	0.111	21.67	2.06	0.189
4-meter walk test (m/s)	-0.022	0.124	-0.016	13.34	1.34	0.312
Handgrip Strength (kg)	0.575	0.294	0.795	13.77	2.19	0.197
Five times sit to stand test (s)	-0.228	0.217	-0.444	15.16	6.01	**0.033**
Women (n=38)						
Fat-Free Mass (kg)	1.190	0.141	0.850	66.67	1.48	0.257
Muscle Mass (kg)	0.414	0.209	-0.081	61.46	0.08	0.802
Appendicular Skeletal Muscle Mass (kg)	0.336	0.223	0.224	71.35	0.90	0.341
Muscle Mass Index (kg/m²)	0.112	0.323	-0.093	55.83	0.92	0.435
Appendicular Skeletal Muscle Index (kg/m²)	0.336	0.223	0.042	70.81	0.30	0.585
Short Physical Performance Battery (score)	-0.098	0.622	-0.149	41.83	0.78	0.531
4-meter walk test (m/s)	-0.015	0.519	-0.015	29.76	0.62	0.613
Handgrip Strength (kg)	-0.043	0.909	0.426	41.64	1.76	0.349
Five times sit to stand test (s)	-0.262	0.558	-0.057	44.88	0.02	0.912

Notes: Adjusted for age, physical activity, protein (g/kg), glomerular filtration rate, allopurinol use and waist circumference.
*R^2% = R^2 value of uric acid plus adjustments minus the R^2 value of the statistical model with only the adjustments variables.

Supplemental Table 3. Characteristics of the participants according to uric acid levels.

	**Normal Uric Acid** *(n=66)*	**Elevated Uric Acid** *(n=47)*
Drugs		
Allopurinol (mg/day)	40.9 ± 55.4	29.8 ± 50.7
Prednisone (mg/ day)	5.7 ± 2.2	6.7 ± 7.2
Tacrolimus (mg/ day)	2.3 ± 3.0	2.7 ± 3.0
Cyclosporine (mg/day)	10.6 ± 32.2	14.4 ± 41.3
Everolimus (mg/day)	0.16 ± 0.43	0.20 ± 0.46
Sirolimus (mg/day)	0.09 ± 0.41	0.0 ± 0.0
Azathioprine (mg/day)	11.1 ± 27.7	5.3 ± 18.7
Mycophenolate sodium (mg/day)	305 ± 424	344 ± 422
Mycophenolate mofetil (mg/day)	310 ± 553	297 ± 577
Loop diuretics (mg/day)	8.6 ± 20.1	18.7 ± 24.5
Thiazide diuretics (mg/day)	1.1 ± 6.8	2.1 ± 8.8

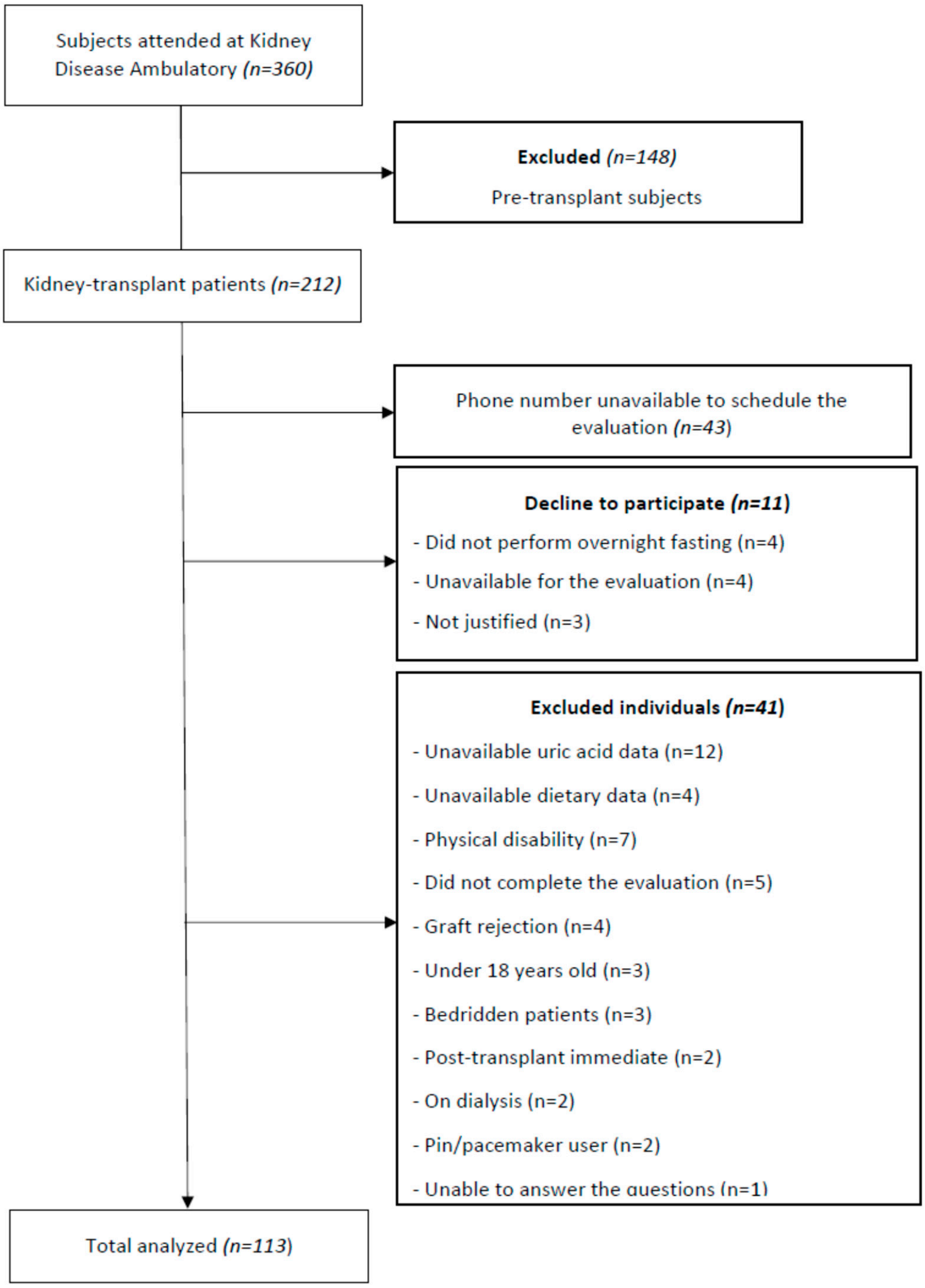

Supplemental Figure 1. Flowchart of the participants.
